# Nucleic Transformer: Classifying DNA Sequences with Self-Attention and Convolutions

**DOI:** 10.1021/acssynbio.3c00154

**Published:** 2023-11-02

**Authors:** Shujun He, Baizhen Gao, Rushant Sabnis, Qing Sun

**Affiliations:** Department of Chemical Engineering, Texas A&M University, College Station, Texas 77840, United States

**Keywords:** genomics, deep learning, natural, language, processing, neural, network, bioinformatics

## Abstract

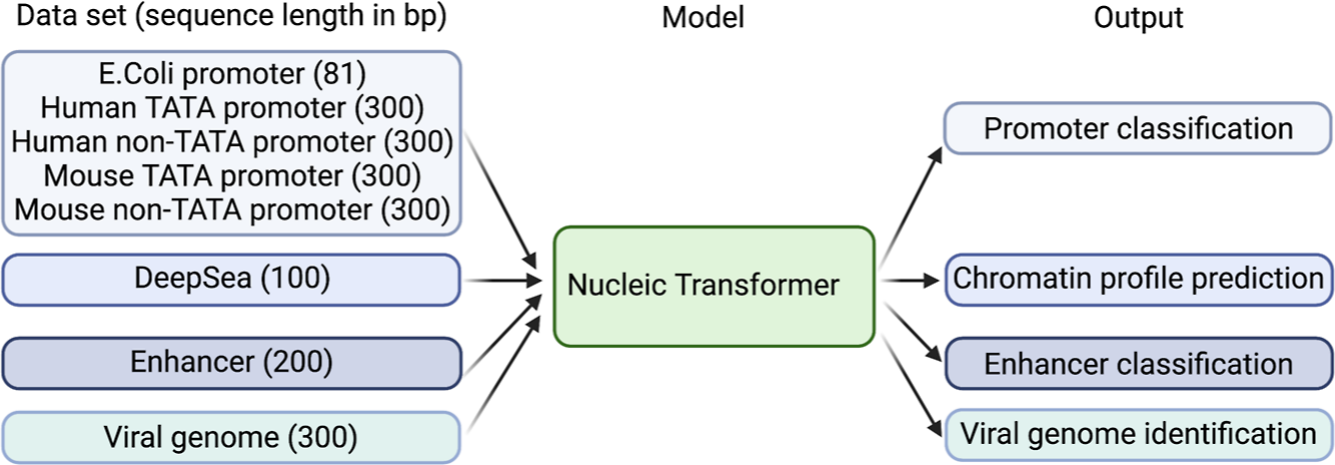

Much work has been done to apply machine learning and deep learning to genomics tasks, but these applications usually require extensive domain knowledge, and the resulting models provide very limited interpretability. Here, we present the Nucleic Transformer, a conceptually simple but effective and interpretable model architecture that excels in the classification of DNA sequences. The Nucleic Transformer employs self-attention and convolutions on nucleic acid sequences, leveraging two prominent deep learning strategies commonly used in computer vision and natural language analysis. We demonstrate that the Nucleic Transformer can be trained without much domain knowledge to achieve high performance in *Escherichia coli* promoter classification, viral genome identification, enhancer classification, and chromatin profile predictions.

## Introduction

DNA sequences are essential components of life. They are associated with almost every biological process for eukaryotes and prokaryotes. Understanding DNA, including the stored information and properties, is critical to understanding life and to reengineering biological systems.^1^ However, DNA tasks have always been challenging because of the complex and large potential sequence space of DNA. Especially, the functions and properties of many coding and noncoding DNA sequences still remain poorly understood.^2,3^

Many techniques have been utilized to extract knowledge from data in the field of bioinformatics with machine learning at the forefront. Machine learning techniques have been successful in leveraging data to reveal underlying patterns, constructing high-performance models, and making accurate predictions. Undoubtedly, many well-known machine learning techniques including random forests, support vector machines, hidden Markov models, Bayesian networks, etc. have been applied in fields such as proteomics, genomics, systems biology, structural biology, and more.^4^ Very recently, deep learning, a subclass of machine learning, has eclipsed traditional machine learning techniques in bioinformatic studies.^5^ Compared to traditional machine learning, deep learning is capable of scaling to much larger amounts of training data, and as a result, providing unparalleled performance; for example, AlphaFold 2 has revolutionized structural biology in the CASP14 challenge, an accomplishment made possible by the power of deep learning.^6^ Further, because biological sequences such as DNA and protein sequences are analogous to natural language, natural language processing (NLP) techniques have been applied to learn from biological data, combining embedding techniques with deep learning.^7,8^

Word embedding techniques have been prominent in the field of NLP, but DNA sequences present a prohibitively large amount of kmers for conventional word embedding techniques, especially when *k* becomes large since the amount of possible kmers scale exponentially with respect to *k*. An alternative is to use one-dimensional (1D) convolution, which has linear complexity with respect to *k*, to represent kmers. Naturally, convolutional networks have been well adopted in the literature,^9−19^ but convolutional networks struggle to grasp extended sequence dependencies vital for DNA-related tasks. Conversely, Transformers employ a neural network design that incorporates self-attention, enabling them to understand relationships across any length of a sequence.^20^ Therefore, a promising idea is to combine self-attention and convolutions to create a neural network that can excel at understanding both short- and long-range interactions present in DNA sequences.

In this study, we present a model architecture Nucleic Transformer that combines convolution and self-attention to understand both local and global dependencies in DNA sequences. Our model achieves high accuracy in a variety of DNA tasks by formulating DNA understanding as NLP tasks; analysis of self-attention weights also provides interpretability. First, the Nucleic Transformer is trained to classify short pieces of DNA sequence (81 bp) as either an *Escherichia coli* promoter sequence or nonpromoter sequence and outperforms other state-of-the-art promoter identification models.^21−23^ In addition to *E. coli* promoters, we also trained Nucleic Transformers that offer superior performance in classifying eukaryotic promoters compared to previous methods.^24^ Second, we show that the Nucleic Transformer achieves better accuracy than DeepSea^17^ in predicting the effects of noncoding variants from 1000 bp fragments. Next, we show that the Nucleic Transformer outperforms a previous top method in enhancer predictions.^25^ Further, the Nucleic Transformer is tested on DNA sequences (300 bp) for classification of viral and nonviral sequences. Lastly, we show that the Nucleic Transformer predicts both viral and nonviral sequences with better accuracy compared with the previous best computational model.^10^

## Results

### Nucleic Transformer Accurately Classifies DNA Promoters and Identifies Consensus Promoter Motifs

We first demonstrate that the Nucleic Transformer outperforms previous models in the literature in *E. coli* promoter classification (Table 1). The Nucleic Transformer beats nondeep learning approaches, which use sophisticated hand-crafted features,^26^ by at least 1.7% or more. A more recent model, iPromoter-BnCNN,^27^ which also employs structural properties of DNA such as stability, rigidity, and curvature, comes close in performance to the Nucleic Transformer, although the Nucleic Transformer directly makes predictions from sequence information. We then confirm the Nucleic Transformer’s superior performance by training with fivefold cross validation 10 times and showing that our Nucleic Transformer demonstrates a statistically significant improvement over iPromoter-BnCNN (Supporting Information Table S4). Further, to compare the transformer encoder with the long short-term memory (LSTM), we swapped out the transformer encoder with a Bidirectional LSTM while keeping all other conditions the same. We see that using the transformer encoder consistently outperforms LSTM on all *k* values (Figure 2a). On the independent test set, which includes recently released experimentally verified promoter samples, the Nucleic Transformer is more accurate than MULTiPly, iPromoter-2L, and iPromoter-BnCNN (Table 2).^22,26,27^ We also constructed additional data sets with more negative samples and show that our Nucleic Transformer remains robust across different levels of class imbalance (Table 3).

**Table 1:** Performance of the Nucleic Transformer in *E. coli* Classification against Top Results in the Literature Based on Accuracy, MCC, Sensitivity, and Specificity

model	accuracy	MCC	sensitivity	specificity
Nucleic Transformer	**0.883**	**0.766**	**0.8832**	0.8832
iPromoter-BnCNN	0.8801	0.7608	0.8643	**0.8959**
MULTiPly	0.8668	0.7224	0.8656	0.8668
iPromoter-2L2.0	0.8498	0.6998	0.8413	0.8584
iPromoter-2L1.0	0.8168	0.6343	0.792	0.8416

**Figure 1:**
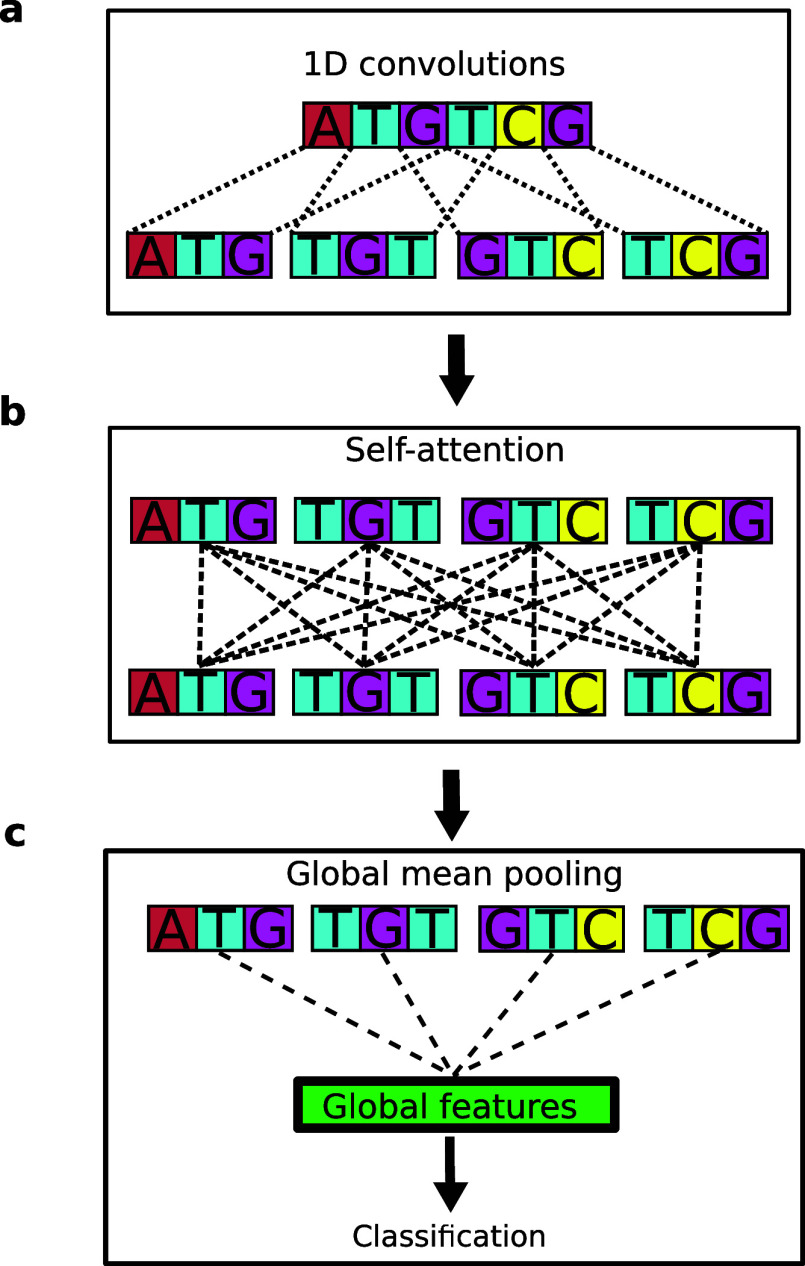
Nucleic Transformer combines self-attention and convolution to learn from DNA data sets. (a) 1D convolutions is used on the nucleotide sequence to segment into kmers. (b) Long-range dependencies are learned by self-attention. (c) Global pooling/deconvolution proceeds the transformer encoder for sequence-level and nucleotide-level predictions.

**Figure 2:**
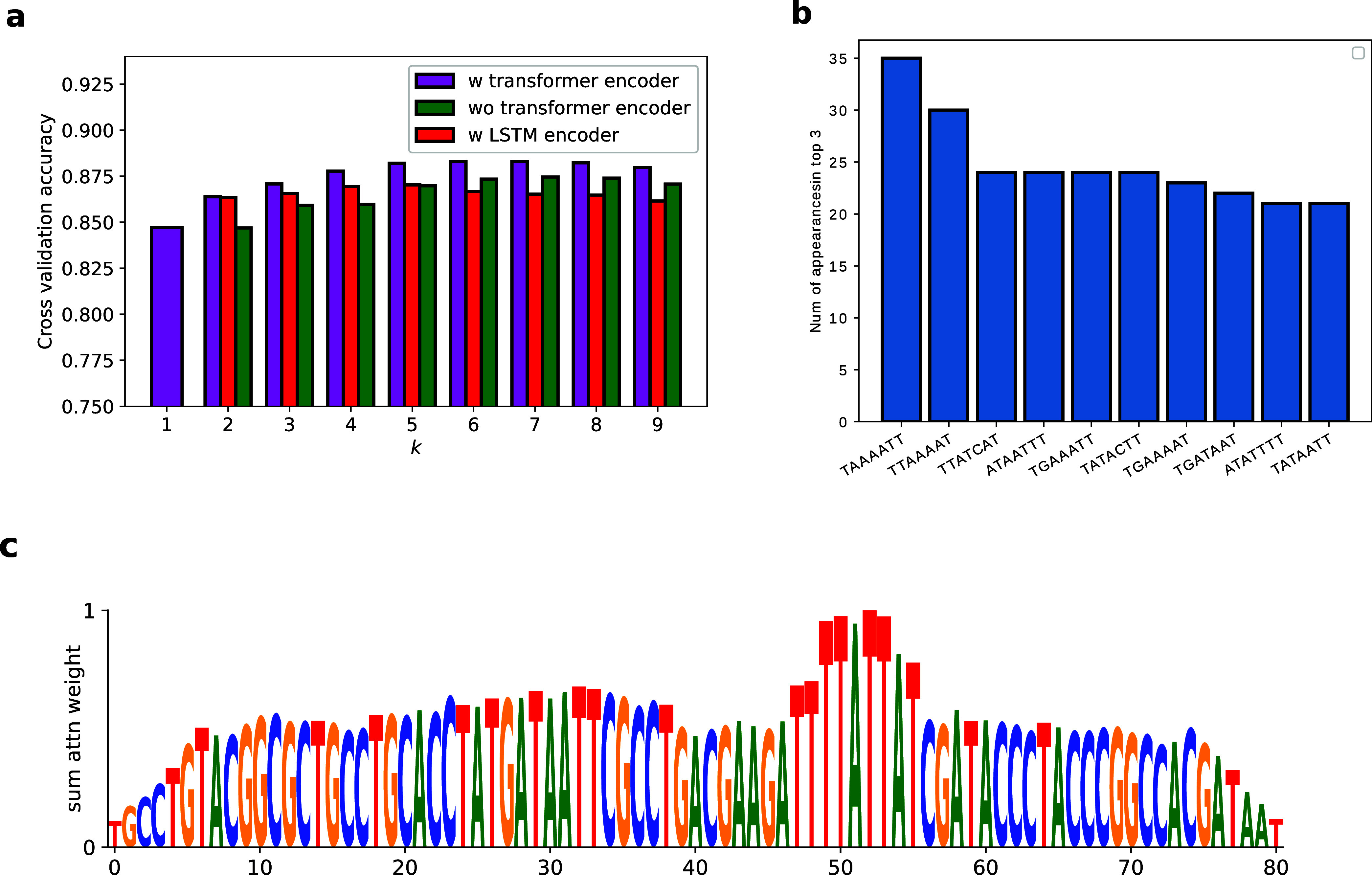
Nucleic Transformer outperforms previous top results in promoter classification and extracts top kmers from data. (a) Bar chart of accuracy vs the parameter *k* of with/without the transformer encoder, and with the LSTM encoder. (b) Most important 7-mers in promoter classification based on the analysis of attention weights. (c) Visualization of an attention weight right before the output of a prediction. The attention weight heavily favors TTATTAT, which is one mutation away from TATAAT, the consensus promoter motif.

**Table 2:** Performance of the Nucleic Transformer in *E. coli* Classification on the Independent Test Set against Top Results in the Literature

model	TP	FN
Nucleic Transformer	**247**	**9**
iPromoter-BnCNN	245	11
MULTiPly	238	18
iPromoter-2L2.0	238	18

**Table 3:** Performance in Promoter Classification with Imbalanced Data Sets of Different Ratios of Promoters vs Non-Promoters^a^

class imbalance	accuracy	sensitivity	specificity	MCC	AUC	AUPR
1–1	0.884	0.898	0.87	0.768	0.947	0.948
1–5	0.927	0.718	0.968	0.724	0.945	0.836
1–10	0.952	0.607	0.987	0.68	0.945	0.768
1–20	0.971	0.535	0.99	0.641	0.947	0.695

a Non-promoters are extracted from the intron and CDS sequences of the *E. coli* k12 genome while excluding sequences with 0.8 redundant sequence identity using CD-HIT.

By visualizing the attention weight matrix, we see that the Nucleic Transformer often focuses on kmers that closely resemble consensus promoter motifs (Figure 2b). We also extracted motifs that the Nucleic Transformer considers the most characteristic of promoters (Figure 2c) and found the kmers that frequently appear in top 3 resemble the consensus promoter motif TATAAT. In fact, one of the 10 most frequently 7-mers have the exact consensus motif TATAAT, while 6 others contain the motif TAAAAT, TATCAT, ATAAT, TATACT, GATAAT, all of which are one mutation away from the consensus motif TATAAT. Deep learning models are hard to interpret due to their complexity and nonlinearity,^28^ but previous efforts have been made to open the black box and recover the feature patterns learned by the model such as DeepLIFT,^29^ which can also be applied to our model and identifies similar motifs as our model (Supporting Information Text S3 and Figure S3). Here, we also demonstrate that the Nucleic Transformer can be interpreted and is learning the right biology.

Apart from *E. coli* promoters, we also explore the performance of the Nucleic Transformer on mouse and human promoters. Previously reported DeePromoter can accurately distinguish between mouse and human (TATA/non-TATA) promoters and promoter sequences with random mutations of segments.^24^ Here, we take a different route in constructing the negative data set by using the flanking regions of promoter sequences, which results in roughly a 2:1 class (nonpromoters to promoters) imbalance. While the Nucleic Transformer outperforms DeePromoter only by a small margin on the classification of TATA promoters of humans and mice, on the more difficult tasks of classifying non-TATA promoters, the Nucleic Transformer leads in performance by large margins [0.22/0.35 Matthews correlation coefficient (MCC)] in human/mouse nono-TATA promoters (Table 4).

**Table 4:** Performance of the Nucleic Transformer across Human and Mouse Species Compared to Previous Results

organism	model	accuracy	precision	recall	F1	MCC
human TATA	DeePromoter	0.9732	0.943	**0.9785**	0.9604	0.94
	Nucleic Transformer	**0.9775**	**0.9621**	0.9706	**0.9663**	**0.9494**
human non-TATA	DeePromoter	0.8373	0.8575	0.9592	0.9055	0.363
	Nucleic Transformer	**0.8829**	**0.9064**	**0.9544**	**0.9298**	**0.585**
mouse TATA	DeePromoter	0.9751	0.9513	0.9779	0.9644	0.9456
	Nucleic Transformer	**0.9776**	**0.958**	0.9779	**0.9678**	**0.9508**
mouse non-TATA	DeePromoter	0.8342	0.8518	**0.954**	0.9	0.4497
	Nucleic Transformer	**0.9015**	**0.9271**	0.9487	**0.9378**	**0.7037**

Previous work in the literature have shown the existence of multiple different functional elements in TATA-less promoters, such as CpG-Islands, G-quadruplexes, and A-tracts.^30−32^ In order to analyze non-TATA promoters, we extracted important 11-mer motifs based on learned model attention from non-TATA human and mouse Nucleic Transformer models (Supporting Information Figures S4 and S5), similarly to *E. coli* promoter motif extraction discussed before. As a result, we see that our model often considers A-tracts important (e.g., AAAAAAAAAAA and CAAAACAAAAC). Further, (partial) G-quadruplexes, which has been shown to be more frequent in TATA-less promoters,^32^ can also be seen (e.g., GGGCGGGCGGG, GGGGAGGGGGAA, and GCCCCTCCCCTC). Unlike our previous analysis of *E. coli* promoters that show highly conserved TATA promoter motifs, we find that important non-TATA motifs are much more diverse.

### Nucleic Transformer Outperforms DeepSea in Chromatin Profile Predictions

**Figure 3:**
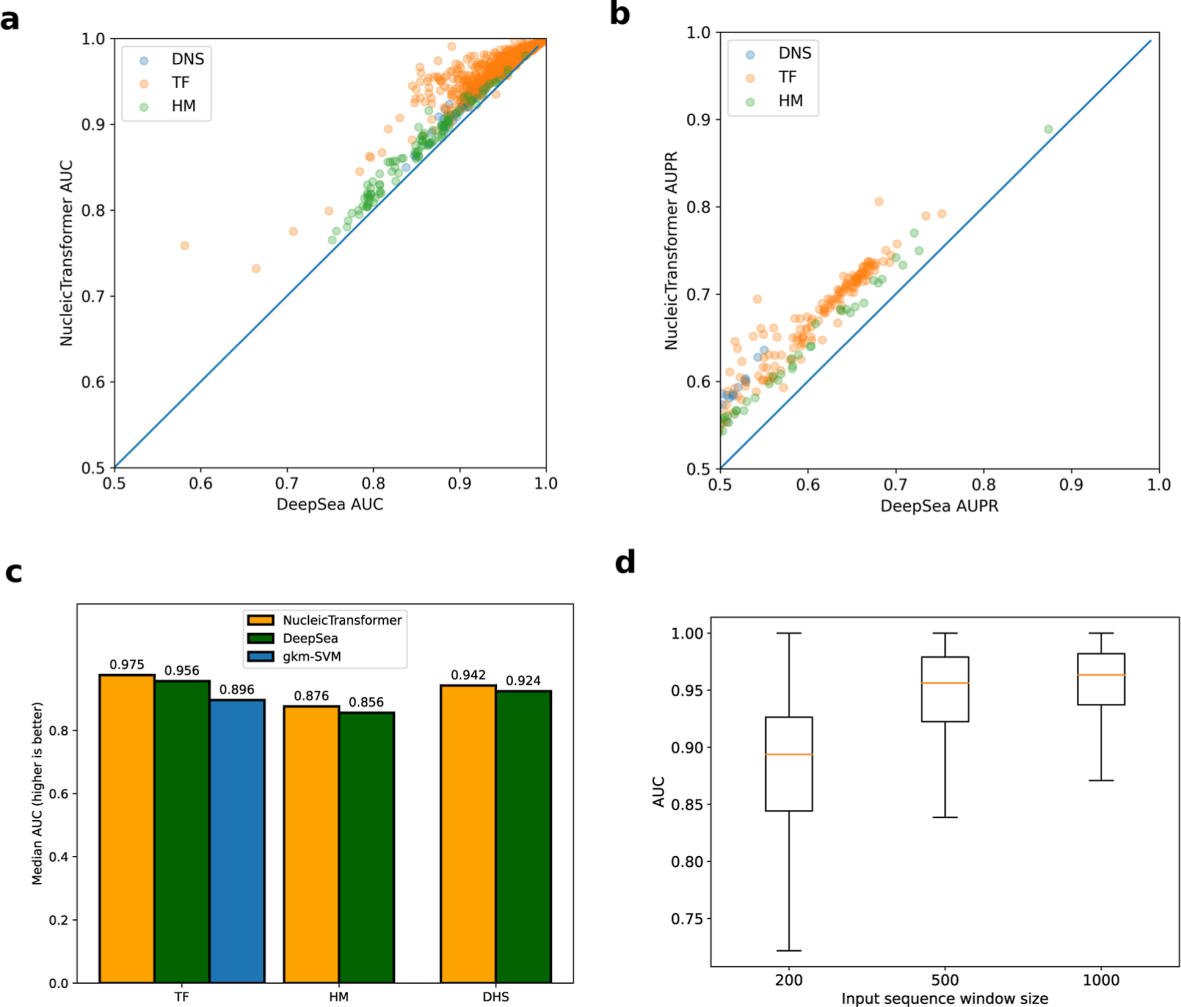
Nucleic Transformer outperforms previous top results in chromatine profile prediction. (a) Test set AUC across 918 classes with positive samples in predicting the effects of noncoding variants comparing Nucleic Transformer and DeepSea. Nucleic Transformer performs significantly better than DeepSea (rank-sum test *p*-value <1.63 10^–30^). The 918 classes are divided into (TF binding), DNSs (DNase I–hypersensitive sites), and HM, and colored accordingly. (b) Test set AUPR across 918 classes with positive samples in predicting the effects of noncoding variants comparing Nucleic Transformer and DeepSea. Nucleic Transformer performs significantly better than DeepSea (rank-sum test *p*-value <1.63 × 10^–30^). (c) Median AUCs in the predictions of TF, DHS, and HM. (d) Nucleic Transformer models with the same architecture as described in the Methods section were trained on 200, 500, and 1000 bp input sequences, respectively, and the AUCs of all chromatin features were shown with box plots. Increasing the context sequence length significantly improved the model performance (*p*-value of <4.10 × 10^–141^ by the Wilcoxon signed rank test between any pair of models.

Identifying the functional effects of noncoding variants is a challenging and important task, since noncoding genomic variations make up the majority of disease and other trait-associated single-nucleotide polymorphisms.^33^ DeepSea has achieved impressive performance with a purely convolution-based deep learning architecture^17^ that significantly outperforms gkm-SVM, a classical machining model that utilizes gapped k-mer features.^34^ To showcase the power of the Nucleic Transformer architecture, we trained Nucleic Transformer models with 919 binary classifiers on the data set used to train DeepSea. In comparison, we see that the Nucleic Transformer demonstrates better AUC and AUPR across almost all 918 classes with positive samples on the test set (Figure 3a,b); in some classes, the Nucleic Transformer shows an AUC improvement of 5–10%. Comparing the median AUC of predicting transcription factors (TF) binding sites, DNase I sensitivity (DHS), and histone mark (HM) profiles (Figure 3c), we see that the Nucleic Transformer holds a sizable advantage over DeepSea in predicting the effects of noncoding variants. Similar to DeepSea, we see a statistically significant increase in model performance when increasing the context length from 200 to 500 and 1000 bp (Figure 3d), demonstrating the Nucleic Transformer’s ability to capture long-range dependencies. Looking at median AUCs of different groups, we see the most substantial increase in performance in HM predictions when training with longer context, consistent with previous reports of HM’s long-range correlations.^35^

### Nucleic Transformer Accurately Identifies DNA Enhancers

While promoters initiate the transcription process, enhancers increase the likelihood of transcription; accurately identifying enhancers is an important task in bioinformatics. Using an enhancer data set previously used to train bert-enhancer,^25^ we trained Nucleic Transformer models to classify enchancers and nonenhancers. We see that the Nucleic Transformer outperforms previous state-of-the-art bert-enhancer by a considerable margin in accuracy and MCC in both cross-validation and independent testing data sets (Tables 5 and 6). While the bert-enhancer also uses convolution, it does so following the bert-encoder. We hypothesize that segmenting DNA sequences into k-mers prior to the transformer network results in more effective learning of long-range interactions between k-mer motifs, as evidenced by the Nucleic Transformer’s superior performance in enhancer classification.

**Table 5:** Cross-Validation Performance on Enchancer Prediction

model	sensitivity	specificity	accuracy	MCC
fastText	0.761	0.744	0.753	0.505
bert-enhancer	**0.795**	0.73	0.762	0.525
Nucleic Transformer	0.779	**0.772**	**0.786**	**0.558**

**Table 6:** Indenpendent Test Set Performance on Enchancer Prediction

model	sensitivity	specificity	accuracy	MCC
EnhancerPred	0.735	0.745	0.74	0.48
iEnhancer-2L	0.71	0.785	0.7475	0.496
iEnhancer-EL	0.71	0.772	0.786	0.46
bert-enhancer	0.8	0.712	0.756	0.514
Nucleic Transformer	**0.81**	**0.75**	**0.78**	**0.561**

### Nucleic Transformer Outperforms Previous Models in Viral Genome Classification

**Figure 4:**
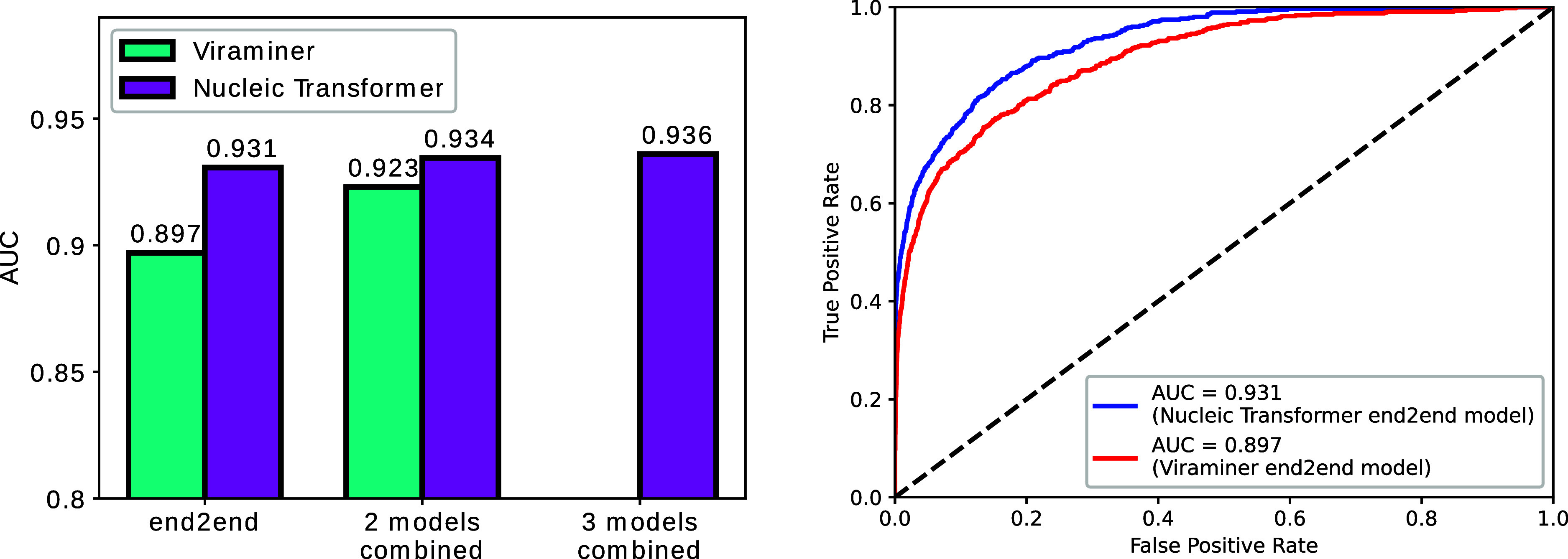
Nucleic Transformer outperforms previous top models in viral genome prediction. (a) Comparison of test set performance between Nucleic Transformer and ViraMiner. (b) ROC AUC curve of ViraMiner versus Nucleic Transformer (best end-to-end models).

To further demonstrate the effectiveness of the Nucleic Transformer architecture, we trained Nucleic Transformer models on a viral/nonviral genome data set previously used to train ViraMiner, a purely convolution-based model,^10^ and compare the performance of the two models. When trained end-to-end, the Nucleic Transformer significantly outperforms the ViraMiner counterpart by a 3.9% area under the ROC curve (AUC) score and a 32% area under the PR curve (AUPR) score (*p*-value of <8 × 10^–5^ using the rank-sum test) (Supporting Information Table S5). When trained with a two-stage training process combining two branches with different hyperparameters and pooling schemes, the ViraMiner performs significantly better compared to its end-to-end counterpart, but the Nucleic Transformer still leads in accuracy even with just one end-to-end model where *k* = 13 (Figure 4a). For better comparison, we also trained the Nucleic Transformer two and three times with different *k*’s and averaged the test set predictions. The AUC improved by 0.3% upon averaging two models (*k* = 11 and 13), but with three models averaged (*k* = 11, 13, and 15), the AUC only improved slightly by 0.2%, which is no surprise since with more models ensembled, there is usually diminishing returns. We also compare the precision recall curve and ROC curve between the best end-to-end models of ViraMiner and the Nucleic Transformer (Figure 4b, Supporting Information Figure S2), and we see that the Nucleic Transformer holds a clear advantage in end-to-end model performance.

### Web Application Development

**Figure 5:**
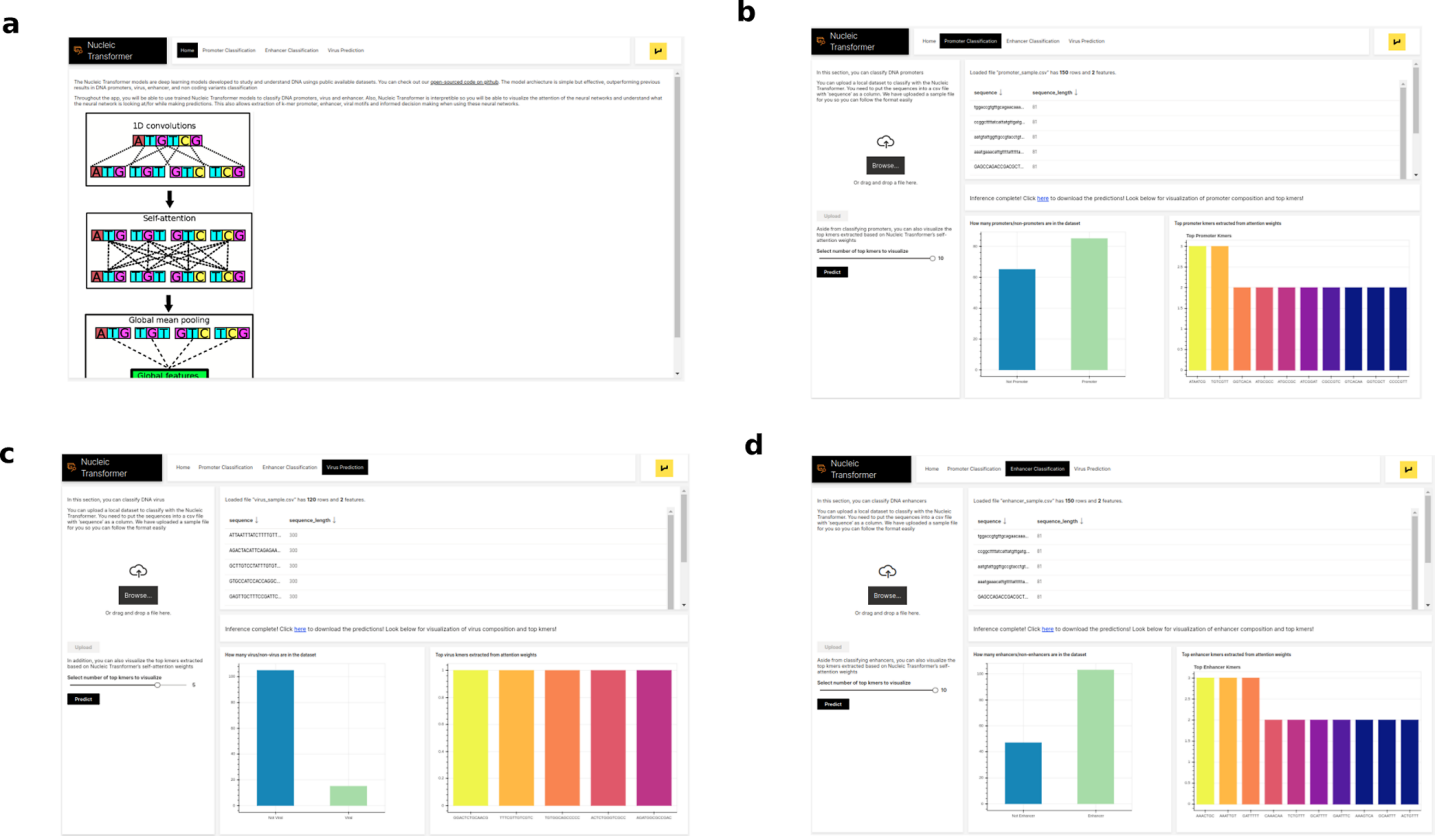
Nucleic Transformer web app. (a) Home page. (b) In this page, the user can classify DNA promoters and visualize the top kmers with pretrained models. (c) In this page, the user can classify DNA promoters and visualize the top kmers with pretrained models. (d) In this page, the user can classify DNA enhancers and visualize the top kmers with pretrained models.

Many previous works on DNA classification have made their models available via web applications.^17,22,26^ These web applications have provided utilities in promoter classification and chromatin profile classification. Here, we also release a web application (Figure 5) made with H2O.ai’s wave, which can be installed from https://github.com/Shujun-He/Nucleic-Transformer-WebApp. Our web app enables not only DNA classification tasks but also visualization of top kilometers based on the Nucleic Transformer’s attention weights. We hope this easy-to-use interface can help biologists adopt our advanced deep learning models.

### Discussion

In this work, we present the Nucleic Transformer, an effective and yet conceptually simple architecture that is capable of high performance in predicting the effects of noncoding variants and classifying promoters, viral genome, and enhancers. The Nucleic Transformer formulates DNA understanding as a NLP task, encoding kmers with 1D convolution and learning long-range interactions with self-attention. We show that the Nucleic Transformer effectively learns from a 1000 bp context and shows improved performance over DeepSea. Further, the Nucleic Transformer architecture outperforms other deep learning/nondeep learning methods that require hand-crafted features in promoter classification while also providing interpretability and being capable of extracting promoter motifs directly from learned attention. Next, the Nucleic Transformer performs better than the previous best model at enhancer predictions. Although always trained end to end, the Nucleic Transformer has better accuracy in classifying viral genomes compared to previous models such as ViraMiner, which requires sophisticated multistage training and ensembling. We demonstrate that the combination of self-attention and convolution is highly effective for tasks in genomics, capturing both broad and specific dependencies; our study corroborates the idea that the transformer can be successful beyond NLP.^36^

## Materials and Methods

### DeepSea Data Set

The DeepSea data set was first compiled by Zhou and Troyanskaya. Training labels were computed from uniformly processed ENCODE and Roadmap Epigenomics data releases. The genomes were split into 200 bp bins, and bins with at least one TF binding event were kept, totaling 512,636,200 bp of sequences. Each training sample was a 1000-bp sequence from the human GRCCh37 reference genome centered on each 200 bp region and was labeled with 919 chromatin features. Training and testing sets were split by chromosomes in a strictly nonoverlapping fashion. Chromosomes 8 and 9 were kept out of the training set to test chromatin feature prediction performances. Area under the receiver operating characteristic curve (AUC) was used to measure performance.

### *E. coli* Promoter/Nonpromoter Data Set

The *E. coli* promoter/nonpromoter data set is an experimentally confirmed benchmark data set widely used in the literature to model and evaluate DNA promoter sequences.^26^ All DNA sequences in the data set were collected from RegulonDB, and sequences were screened by CD-HIT based on redundant sequence identity.^37^ This data set consists of 2860 promoter sequences and 2860 nonpromoter sequences. All promoter sequences were experimentally confirmed and collected from RegulonDB (version 9.3).^38^ The nonpromoter sequences were extracted randomly from the middle regions of long coding sequences and convergent intergenic regions in the *E. coli* K-12 genome.^39,40^ Model performance on this data set was evaluated using fivefold cross-validation, where the data were split using iterative stratification.^41^ The metrics used for this data set are accuracy, sensitivity, specificity, and MCC. In addition to cross-validation, we also used an independent test set composed of experimentally verified *E. coli* promoters recently added to RegulonDB. Lastly, we extracted additional nonpromoter sequences (filtered by CD-HIT by 0.8 redundant sequence identity) from the intron and CDS regions of the *E. coli* genome and constructed data sets with 5:1, 10:1, and 20:1 imbalance.

### Human and Mouse Promoter Data Sets

The TATA/non-TATA human and mouse promoter sequences were downloaded from https://epd.epfl.ch//index.php.^42,43^ Specifically, for each promoter sequence, we downloaded the −549 to 350 bp region so we can use the middle −249 to 50 bp regions as positive promoter examples and the two flanking regions as negative examples. Subsequently, positive and negative sets were screened by CD-HIT based on redundant sequence identity using a threshold of 0.8.^37^ Note that since two flanking regions were extracted per promoter sequence, these data sets have roughly twice the number of nonpromoters as promoters.

### Enhancer/Nonenhancer Data Set

The enchancer/nonenhancer data set was first introduced in the iEnhancer-2L study.^44^ Enhancer sequences from nine different cell lines were collected and segmented into 200 bp fragments. With CD-HIT,^37^ DNA sequences were filtered to exclude the ones with high (>20%) similarity, resulting in 1484 enhancer sequences and 1484 nonenhancer sequences to use for training and validation and 200 enhancer sequences and 200 nonenhancer sequences to use for testing.

### Viral/Nonviral Genome Data Set

The viral/nonviral genome data set is same as the one used to train ViraMiner,^10^ which consists of 19 different NGS experiments analyzed and labeled by PCJ-BLAST^45^ following de novo genome assembly algorithms. This data set is publicly available at https://github.com/NeuroCSUT/ViraMiner. DNA sequences included in this data set were cut to 300 bp segments, with the remaining portions smaller than 300 bp discarded. Further, all sequences that contain “*N*” (unknown with equal probability to any of the four nucleotides) were removed as well. This data set has approximately 320,000 DNA sequences in total. The main challenge with this data set is the class imbalance, where only 2% of sequences are of viral origin. The data set is split into training, validation, and test, where hypertuning was done with the training and validation set and the model performance was evaluated on the test set. The metric used for this data set is AUC.

### Nucleic Transformer Combines Self-Attention and Convolution to Learn from DNA Data Sets

Any DNA sequence is a series of nucleotides, each of which can be one of A (adenosine), C (cytidine), G (guanosine), and T (thymine). Therefore, if we consider DNA to be a language that uses only four different characters to encode information, we can model it in a similar fashion as a natural language. This idea then allows us to agnostically apply NLP techniques in the domain of deep learning without injection of domain-specific knowledge in biology. Take the English language for example; all words in the vocabulary are combinations of the 26 letters in the alphabet; similarly, a DNA sequence is a sequence of 4 nucleotides. Although there are similarities between DNA sequences and natural language, there are also some important differences. The English language not only contains letters but also spaces that separate words and commas and periods that separate sentences, whereas comparatively, a DNA sequence is simply a sequence of 4 nucleotides. Further, when humans interpret a sentence, words are discretely recognized, and each word can be considered a discrete object. As a result, state-of-the-art NLP models evaluate languages as collections of words (and punctuations) instead of letters. Since a DNA sequence does not have punctuations, we need to find a way to transform a DNA sequence into “words”. To do this, we transform the DNA sequences into kmers with 1D convolutions (Figure 1a) following a single nucleotide embedding layer, a deep learning operation that allows for efficient and effective motif recognition
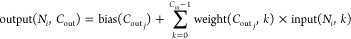
1where input is the single nucleotide embeddings, *C*_in_ and *C*_out_ are the input and output channels, and *N* is the batch size.

The intuition behind 1D convolutions (Figure 1a) is due to its conceptual correspondence with kmer analysis/features, which is a method for analyzing DNA sequences by dividing them into short sequences of k nucleotides, called k-mers. Extracting k-mers from a DNA sequence is akin to using a sliding window of size k, capturing segments of the sequence as it shifts one step at a time from start to finish. This method mirrors the convolution technique in deep learning and has also been employed in predictions related to RNA degradation rates as well.^46^ Here, 1D convolution allows us to have learnable kmer representations as input to the subsequent transformer network, which learns global dependencies.

Although effective at motif recognition, convolution on its own cannot capture long-range dependencies that are prevalent in DNA. Other works in the literature have applied recurrent neural networks such as LSTM networks, but LSTM’s sequential nature means that it is still insufficient at modeling relationships at long distances, as demonstrated by the transformers’ superior performance at machine translation and natural language understanding.^20,48^ To enable our neural network to learn long-range dependencies at any distance, we used transformer encoder layers with self-attention following the 1D convolution layer (Figure 1b). We also provide a table outlining the difference between our approaches and other methods in this study (Supporting Information Table S2). Multiheaded self-attention networks, which process the entire sequence altogether, allow for faster computation compared to other sequence processing methods such as recurrent neural networks and also perform better at capturing long-range dependencies
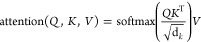
2
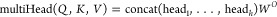
3
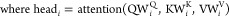
4where *Q*, *K*, and *V* are kmer representations outputted by 1D convolution,  is the size of the attention head, *W*^O^ is the linear transformation matrix for the concatenated attention head outputs, and *W*_*i*_^*Q*^, *W*_*i*_^*K*^, and *W*_*i*_^*V*^ are the linear transformation matrices for *Q*, *K*, and *V* before the *i*th attention head, respectively. The self-attention function is followed by a positionwise feedforward network applied separately and identically to each position
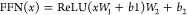
5where *W*_1_ and *W*_2_ are linear transformation matrices, and *b*_1_ and *b*_2_ are bias matrices for the two linear transformations, respectively.

### Global Pooling

Following the transformer encoder layers, we use one global pooling layer right before making predictions appropriate for the task (Figure 1c)
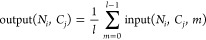
6where *l* is the length of the input sequence, *N* denotes the batch dimension, and *C* denotes the channel dimension. Combining 1D convolutions and self-attention makes our neural networks adept at modeling both local motifs and long-range dependencies, which are two key aspects of modeling DNA.

### Positional Encoding

Self-attention is permutation invariant, so we use sinusoidal positional encoding to represent the positions of DNA nucleotides^20^
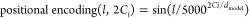
7
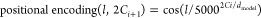
8where *l* is the position and *C* is the channel dimension, and *d*_model_ is the size of the embeddings.

### Optimizer and Training Schedule

We use Adam,^49^ a commonly used optimizer in deep learning with β_1_ = 0.9, β_2_ = 0.99, and ϵ = 1*e* – 8. Weight decay is set to 1 × 10^–5^. Our learning rate schedule is a stepwise inverse square root decay schedule with warmup. Since we use relatively small batch sizes during training, we adjust the learning rate by a scaling factor *C*
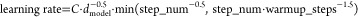
9

In our experiments, *C* is set to 0.1, and warmup_steps is set to 3200. Additionally, we use dropout^50^ of probability 0.1 in all attention layers, fully connected layers, and positional encoding.

### Random Mutations during Training

It is known that deep learning models can perfectly memorize completely random data and labels, so to combat the memorization effect, we inject noise artificially by randomly mutating positions in the source DNA sequence before forward and backward propagation during training, similar to bert’s pretraining.^48^ This injection of random noise is done during DNA supervised learning. Note that in all our experiments, we simply randomly mutate nucleotides in randomly selected positions, and because we do not ensure the nucleotide at each selected position is changed, the average amount of mutations is 3/4 of *n*_mute_. It is true that these random mutations could be nonlabel-preserving; however, the deep learning algorithm is robust to massive label noise, so the nonlabel-preserving mutations should simply be ignored by the network during training.^51^ The number of positions to randomly mutate is a hyperparameter that we denote as *n*_mute_, with which we experiment to find the best value.

### Best Hyperparameters for Different Tasks

For *E. coli* promoter classification, the best results were obtained using *k* = 7, *n*_mute_ = 15, six transformer encoder layers, *d*_model_ = 256, *n*_head_ = 8, and a batch size of 24. For human and mouse promoters, the best results were obtained using *k* = 11, *n*_mute_ = 45, six transformer encoder layers, *d*_model_ = 256, *n*_head_ = 8, and a batch size of 64. For the DeepSea data set, we introduced two more Conv 1D layers and three maxpooling layers to reduce the input sequence length to transformer encoder; the best results were obtained using an ensemble of *k* = 7 and *k* = 9, *d*_model_ = 1024, and *n*_head_ = 16. For enchancer predictions, the same hyperparamters as those for *E. coli* promoters were used. For viral/nonviral DNA classification, the best results were obtained using *n*_mute_ = 40, six transformer encoder layers, *d*_model_ = 512, and *n*_head_ = 8. For all binary classification tasks, we used a cutoff of 0.5 to determine positive vs negative predictions.

## Code Availability

All training code to fully reproduce results is released at https://github.com/Shujun-He/Nucleic-Transformer, and a web application (Figure 5) developed using H2O.ai’s wave is available at https://github.com/Shujun-He/Nucleic-Transformer-WebApp.

## Supporting Information Available

Tuning of hyperparameters, Nucleic Transformer performance across different levels of class imbalance, and extraction of promoter motifs (PDF)
