# Supplementary material for: Nucleic Transformer: Classifying DNA Sequences with Self-Attention and Convolutions
Source: ACS Synth Biol. 2023 Nov 2;12(11):3205–14. doi: 10.1021/acssynbio.3c00154 (PMC10863451; doi:10.1021/acssynbio.3c00154)
Supplement: Supplementary file 1 — sb3c00154_si_001.pdf [file sb3c00154_si_001.pdf]

Supplementary information

1 Tuning of hyperparameters

Our approach began by selecting an initial set of hyperparameters based on experience. We then conducted experiments with various factors, such as batch size and epochs trained. For each hyperparameter, we individually tested different values (e.g., 8, 16, 32 for batch size) and retained the best-performing one while evaluating the rest. Compared to grid search, which requires testing many more hyperparameter combinations, our method proves to be significantly more efficient. For example, if we were to examine three different hyperparameters, each with three values, our approach would entail conducting individual tests three times, resulting in a total of nine parameter sets (3+3+3). In contrast, grid search would necessitate examining 27 combinations (3x3x3). Thus far, our findings indicate that 'k' has the most substantial impact on performance, because larger k-mer convolutions enable the model to learn more high-level interactions, though there is a potential risk of overfitting.

2 Nucleic Transformer remains robust across different levels of class imbalance

Although typically in machine learning datasets with a class balance are constructed to test model performance, in reality promoter sequences are much more scarce compared to non-promoter sequences. To account for this, we additionally constructed 3 more datasets with more non-promoter sequences (5x, 10x, 20x non-promoter sequences) from the intron/CoDing Sequence(CDS) region of the *E. coli* genome. After training on the additional imbalanced datasets, we see that the Nucleic Transformer has almost the same AUC score across different levels of class imbalance (**??**), indicating its robustness despite class imbalance.

3 Extraction of promoter moifs

For each correct prediction of promoters in the validation sets of 5-fold cross validation, we take the column-wise sum of the attention weight matrix and rank the kmers. Then we simply count the amount of times each kmer receives top 3 most attention in all correct predictions. Aside from extraction using attention weight, we also experimented with DeepLIFT [**?**] (rescale rule). We applied different baselines using the rescale rule and found overall they give similar results to our simple attention weight analysis (**??**).

Bibliography

[] Avanti Shrikumar, Peyton Greenside, and Anshul Kundaje. Learning important features through propagating activation differences. *CoRR*, abs/1704.02685, 2017. URL http://arxiv.org/abs/1704.02685.

Dataset	Training set size	Testing set size	Sequence Length	Percent Positive
DeepSea	4440000	8000	1000	0.022
E.Coli promoter	5720	256	81	0.5
Human TATA promoter	5277	1319	300	0.33
Human non-TATA promoter	18800	6267	300	0.33
Mouse TATA promoter	5367	1789	300	0.33
Mouse non-TATA promoter	16137	5380	300	0.33
Enhancer	3578	400	200	0.5
Viral genome	211236	26405	300	0.02

Supporting Information Table S1: Summary of datasets used in this study.

Method	Uses features	Convolution	LSTM	Self-attention	Interpretability
Nucleic Transformer	No	Yes	No	Yes	Yes
Ipromoter-BnCNN	Yes	Yes	No	No	Yes
DeePromoter	No	Yes	Yes	No	No
DeepSea	No	Yes	No	No	No
Bert-enhancer	No	Yes (2D convolution)	No	Yes	No
Viraminer	No	Yes	No	No	No

Supporting Information Table S2: Comparison of our model with other methods in the literature.

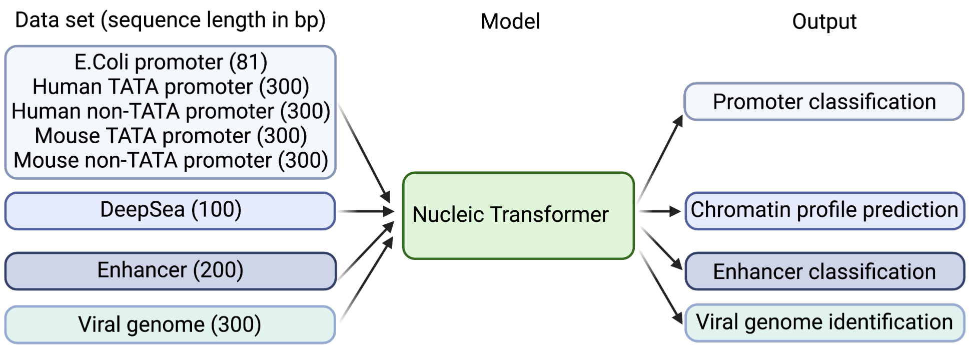

Supporting Information Figure S1: Overview flow chart.

class imbalance	accuracy	sensitivity	specificity	MCC	AUC	AUPR
1-1	0.884	0.898	0.87	0.768	0.947	0.948
1-5	0.927	0.718	0.968	0.724	0.945	0.836
1-10	0.952	0.607	0.987	0.68	0.945	0.768
1-20	0.971	0.535	0.99	0.641	0.947	0.695

Supporting Information Table S3: Performance in promoter classification with imbalanced datasets of different ratios of promoters vs non-promoters. Non-promoters are extracted from the intron and CDS sequences of the *E. coli* k12 genome while excluding sequences with 0.8 reduant sequence identify using CD-HIT.

	Nucleic Transformer	iPromoter BCNN
ACC	0.883±0.0015	0.8801±0.0032
sensitivity	0.8832±0.0093	0.8643±0.0106
spec	0.8827±0.0083	0.8959±0.0118
MCC	0.766±0.003	0.7608±0.0065

Supporting Information Table S4: We compare Nucleic Transformer and iPromoter BCNN on the *E. coli* dataset by training them 10 times and averaging cross validation metrics. Nucleic Transformer outperforms iPromoter BCNN significantly in accuracy (p-value=0.0046 using ranksum test).

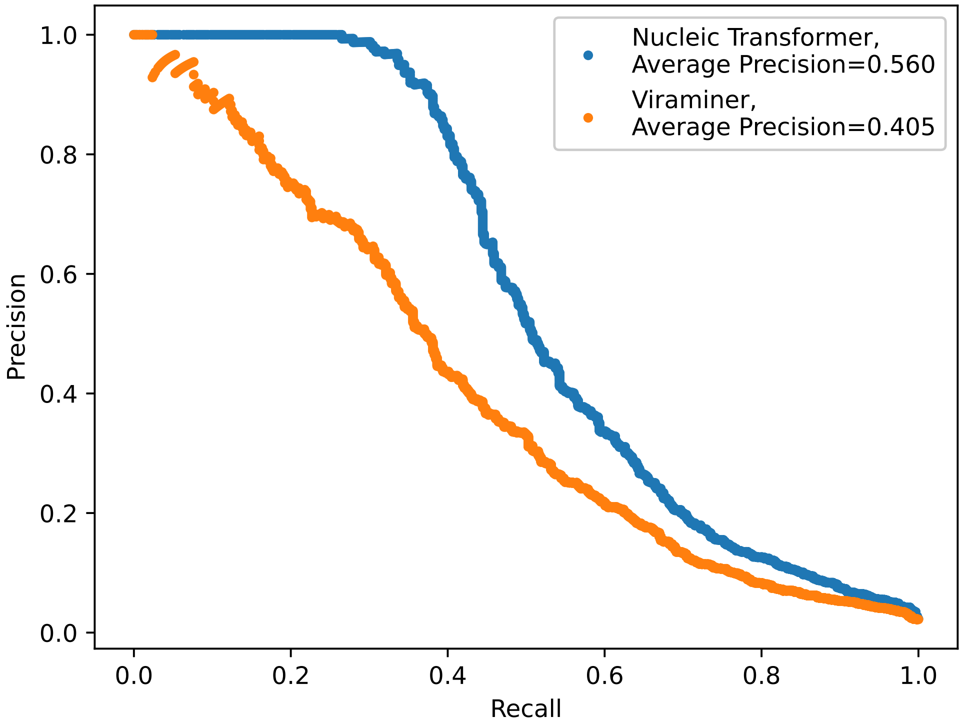

Supporting Information Figure S2: Precision-recall curve of viraminer vs Nucleic Transformer (best end-to-end models)

Method	AUROC	AUPR
Nucleic Transformer	**0.9192±0.0064**	**0.5487±0.0086**
Viraminer	0.8801±0.0082	0.3282±0.0285

Supporting Information Table S5: Statistical test of Viraminer vs Nucleic Transformer (end-to-end). Nucleic Transformer showed significant better performance (ranksum test p-value¡8e-5, N=10)

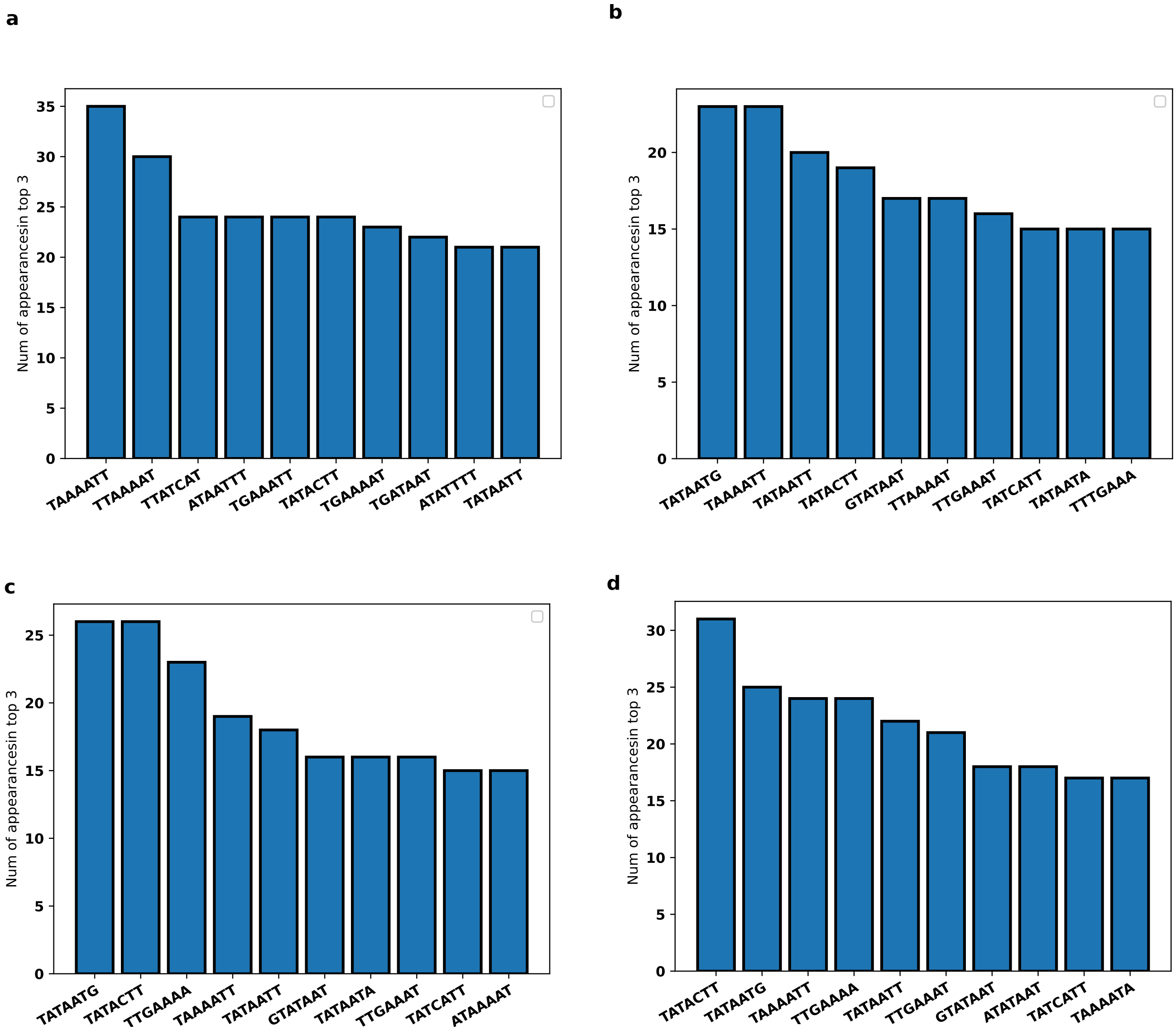

Supporting Information Figure S3: **We extracted *E. coli* motifs using DeepLIFT (rescale rule) to compare with our attention weight motif extraction. DeepLIFT produced similar results with different baselines, with 3 to 4 top motifs coinciding with the ones extracted directly from attention weights and most of them resembling consensus promoter motif TATAAT.** **a.** Motifs extracted directly from attention weights. **b.** Motifs extracted with DeepLift with random sequences (10 times and averaged) as baselines. **c.** Motifs extracted with DeepLift with random permutation of original sequences (10 times and averaged) as baselines. **d.** Motifs extracted with DeepLift with 0 values as baselines.

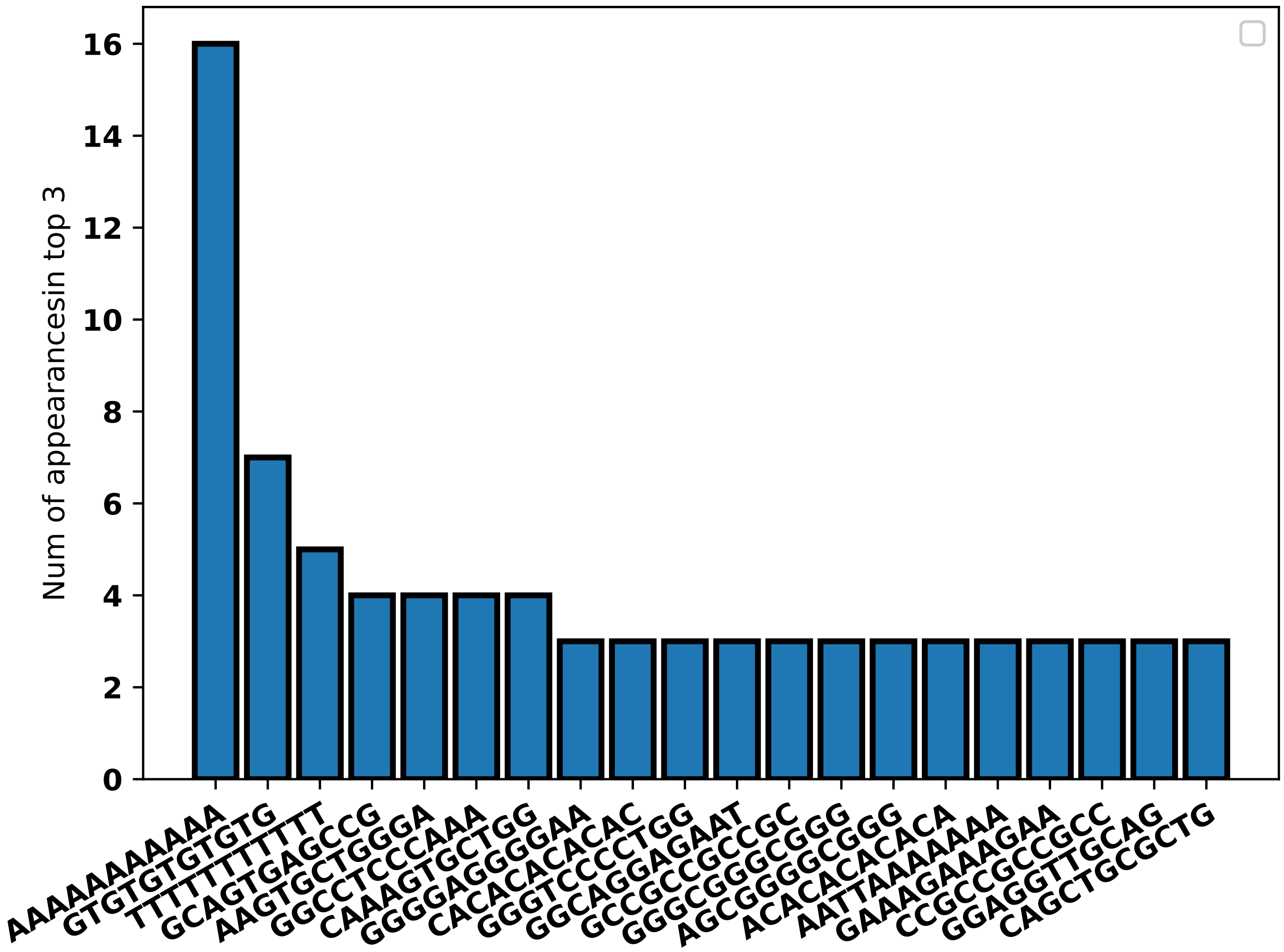

Supporting Information Figure S4: Extracted human non-TATA promoter motifs based on learned attention

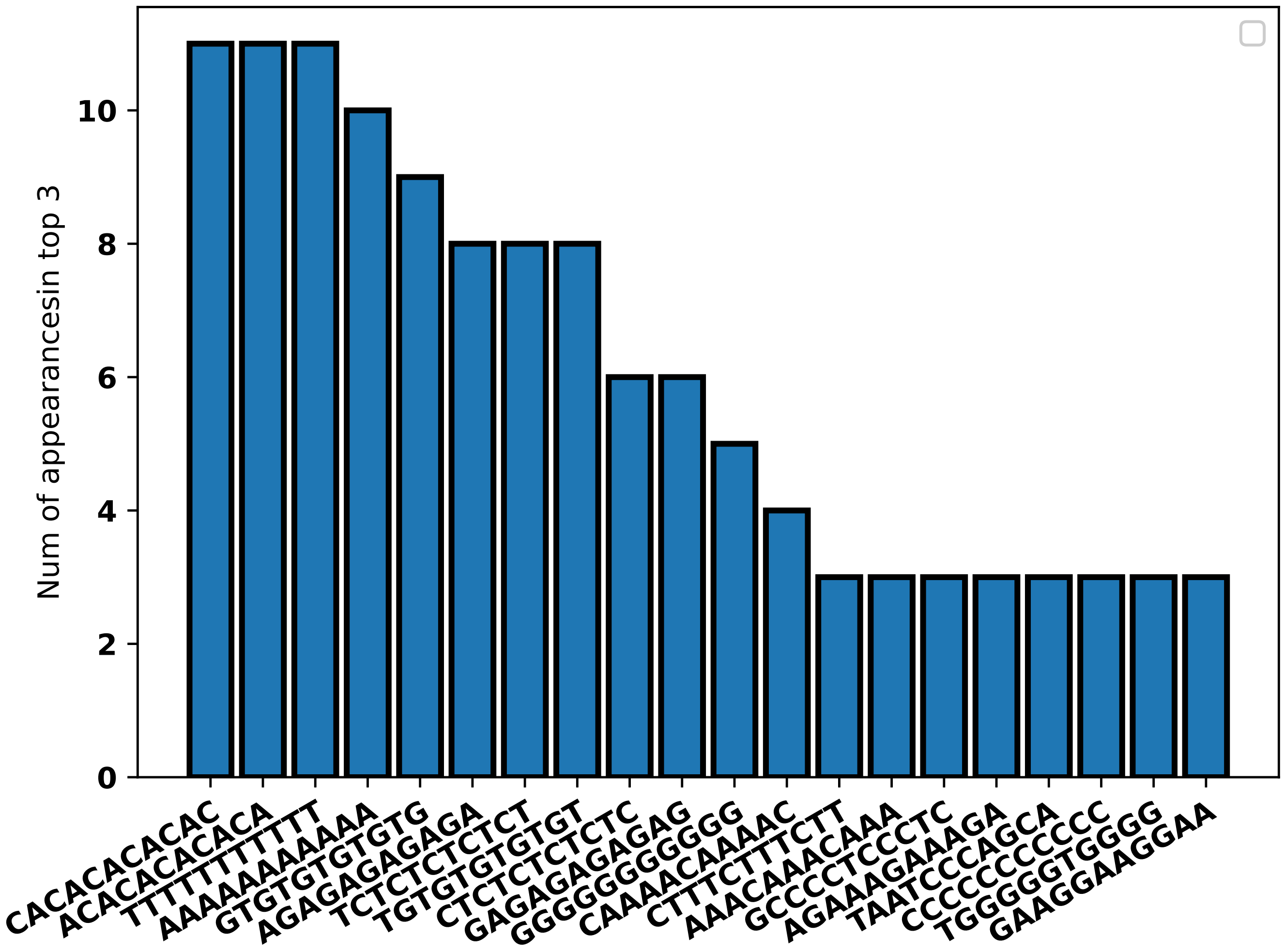

Supporting Information Figure S5: Extracted mouse non-TATA promoter motifs based on learned attention

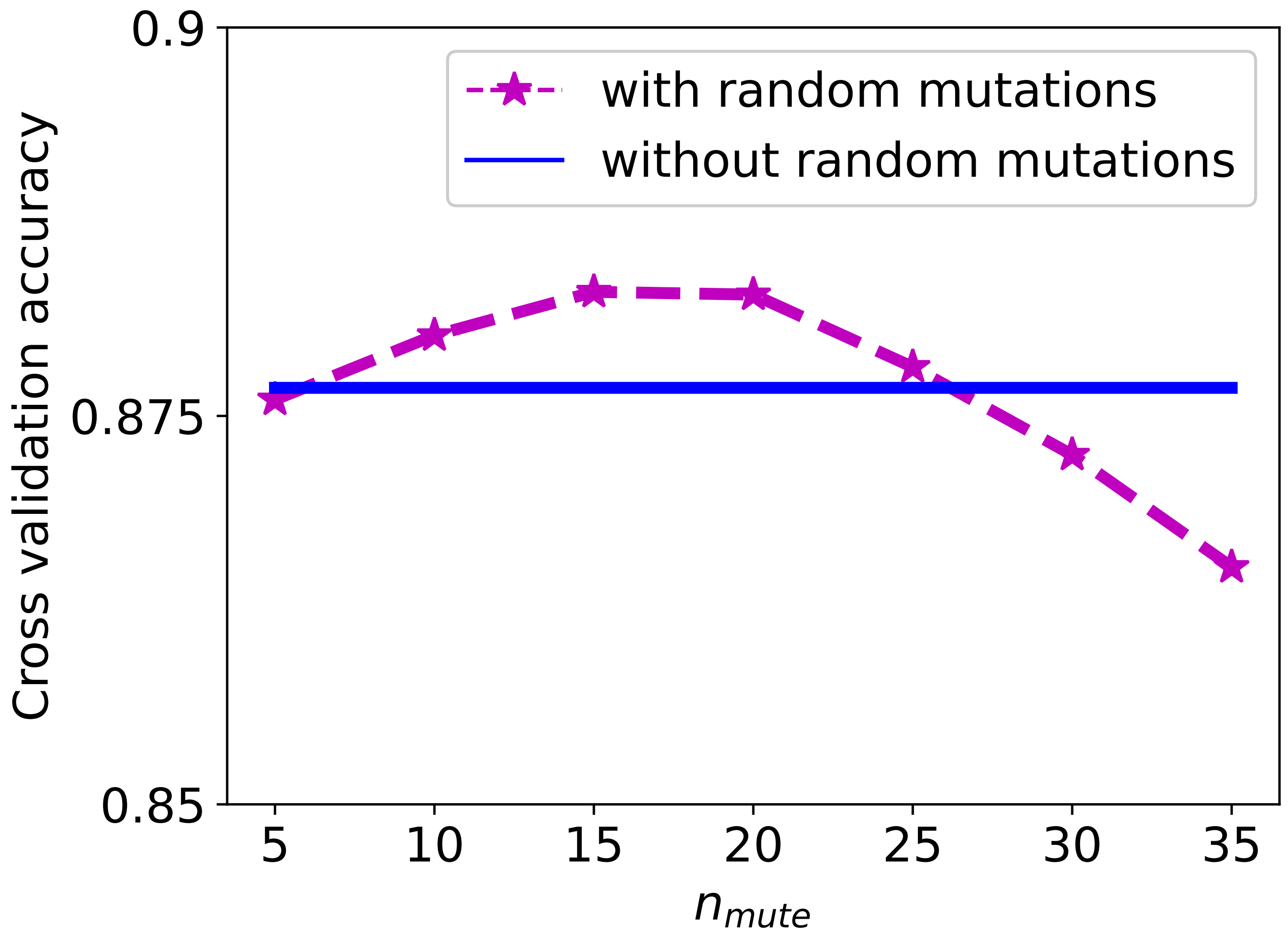

Supporting Information Figure S6: Effect of random mutations during training on E.Coli promoter dataset. We varied number of random mutations and observed cross validation accuracy.
